# Supplementary material for: Manganese Sulfanyl Porphyrazine–MWCNT Nanohybrid Electrode Material as a Catalyst for H2O2 and Glucose Biosensors
Source: Sensors (Basel). 2024 Sep 27;24(19):6257. doi: 10.3390/s24196257 (PMC11478948; doi:10.3390/s24196257)
Supplement: Supplementary file 1 [file sensors-24-06257-s001.zip › sensors-3183037-supplementary.pdf]

Supplementary data

For

Manganese sulfanyl porphyrazine–MWCNT nanohybrid electrode material as a catalyst for H_2O_2 and glucose biosensors

Michal Falkowski [1,2]*, Amanda Leda [3], Mina Hassani [1], Michal Wicinski [4], Dariusz T. Mlynarczyk [5], Nejat Düzgüneş [6], Michal P. Marszall [1], Grzegorz Milczarek [3], Jaroslaw Piskorz [7], Tomasz Rebis [3]*

[1] Department of Medicinal Chemistry, Collegium Medicum in Bydgoszcz, Faculty of Pharmacy, Nicolaus Copernicus University in Torun, Dr. A. Jurasza 2, 85-089 Bydgoszcz, Poland; 503341@doktorant.umk.pl (M.H.); mmars@cm.umk.pl (M.P.M.)
[2] Faculty of Health Sciences, Collegium Medicum, The Mazovian University in Płock, 09-402 Płock, Poland
[3] Institute of Chemistry and Technical Electrochemistry, Poznan University of Technology, Berdychowo 4, 60-965 Poznan, Poland; amanda.leda@doctorate.put.poznan.pl (A.L.); grzegorz.milczarek@put.poznan.pl (G.M.)
[4] Department of Pharmacology and Therapy, Collegium Medicum in Bydgoszcz, Faculty of Medicine, Nicolaus Copernicus University in Torun, Curie Sklodowskiej 9, 85-094 Bydgoszcz, Poland; michal.wicinski@cm.umk.pl (M.W.)
[5] Chair and Department of Chemical Technology of Drugs, Poznan University of Medical Sciences, Rokietnicka 3, 60-806 Poznan, Poland; mlynarczykd@ump.edu.pl (D.T.M.)
[6] Department of Biomedical Sciences, Arthur A. Dugoni School of Dentistry, University of the Pacific, San Francisco, CA 94103, USA; nduzgunes@pacific.edu (N.D.)
[7] Chair and Department of Inorganic and Analytical Chemistry, Poznan University of Medical Sciences, Rokietnicka 3, 60-806 Poznan, Poland; piskorzj@ump.edu.pl (J.P.)

* Correspondence: m.falkowski@cm.umk.pl (M.F.); tomasz.rebis@put.poznan.pl (T.R.)

Table of Contents

1. HPLC purity of porphyrazine (3)....................2

2. Electrochemical studies....................7

Figure S1. CVs of GC (A), and GC/MWCNT (B) electrodes. An experiment was recorded in PB (pH 7.4) before (a, marked by black line) and after (b, marked by blue line) 2 mM H_2O_2 addition. Scan rate 10 mV s^{-1}.

Figure S2. The results of cyclic voltammetry studies of (A) GC, (C) GC/MWCNT, (E) GC/MWCNT/Pz3, and (G) GC/MWCNT/Pz3/GOx in phosphate buffer (pH 7.4) containing 1 mM $K_3[Fe(CN)_6]$ at the scan rate range 0.01–0.05 V s^{-1}. (B, D, F, H) Change in peak cathode currents as a function of the applied square root of the scan rate for appropriate electrodes.

Figure S3. Chronoamperometric response to the addition of 1 mM Glu for the GC/MWCNT/GOx (a, marked by black line), and GC/MWCNT/Pz3/GOx (b, marked by blue line) electrode at an applied potential of +0.6 V.

Figure S4. Cyclic voltammograms of the 7 newly prepared GC/MWCNT/Pz3/GOx electrodes recorded in PB buffer at 50 mV s^{-1}. RSD is equal 4.6% (n = 7).

Figure S5. Stability of the GC/MWCNT/Pz3/GOx biosensor to a 1 mM Glu response recorded over time. with an applied potential of +0.6 V.

Figure S6. Chronoamperometric responses recorded at the GC/MWCNT/Pz3/GOx biosensor upon the addition of glucose and selected interferents with an applied potential of +0.6 V. The supporting electrolyte: phosphate buffer (PB, pH 7.4).

Figure S7. (A) Chronoamperometric curve recorded at +0.6 V in PB after additions of real sample and 1 mM glucose standard solutions. (B) Standard addition curve corresponding to curve presented in (A). RSD = 0.7% (n = 3).

1. HPLC purity

The HPLC analysis using an Agilent 1200 instrument equipped with a UV-DAD detector was utilized to assess the purity of macrocycle **3**. An octadecylsilane-coated column (Eclipse XDB-C18, 150 mm × 4.6 mm, 5 μm, Agilent) was used for chromatographic separations with gradient elution conditions at a 1 mL/min flow rate. Band dispersion and additional peaks from aggregates significantly hampered HPLC analysis. The best conditions for the compounds are shown below.

Manganese(III) porphyrazine (3)

Configuration 1

Phase		
**time [min]**	**dichloromethane**	**methanol**
0	100	0
3	100	0
4	75	25
15	75	25

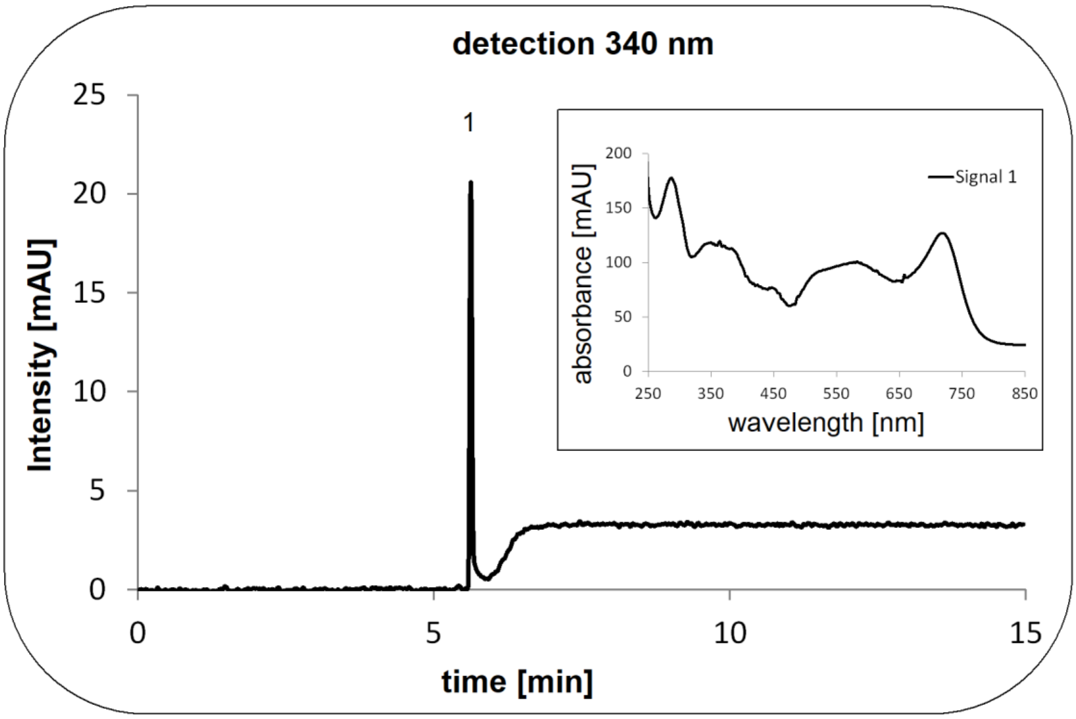

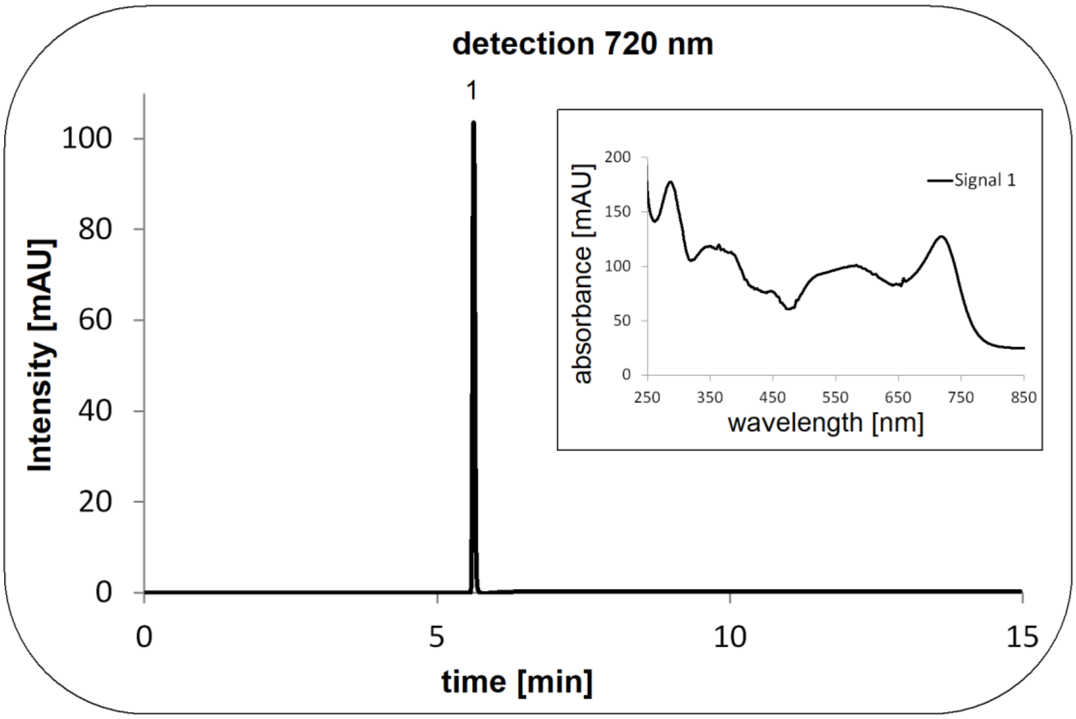

Results			
signal	retention time [min]	area	purity [%]
detection 340 nm			
1	5.6	59.7	100.0
detection 720 nm			
1	5.6	104.5	100.0

Configuration 2

Phase		
time [min]	dichloromethane	2-propanol
0	100	0
3	100	0
4	40	60
15	40	60

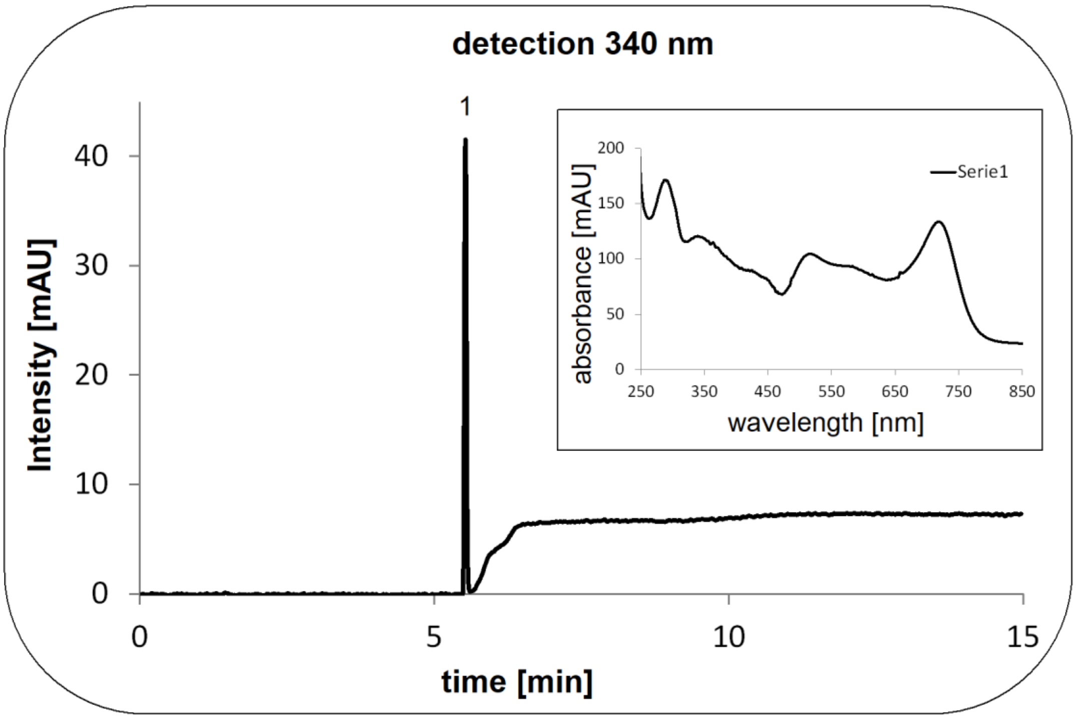

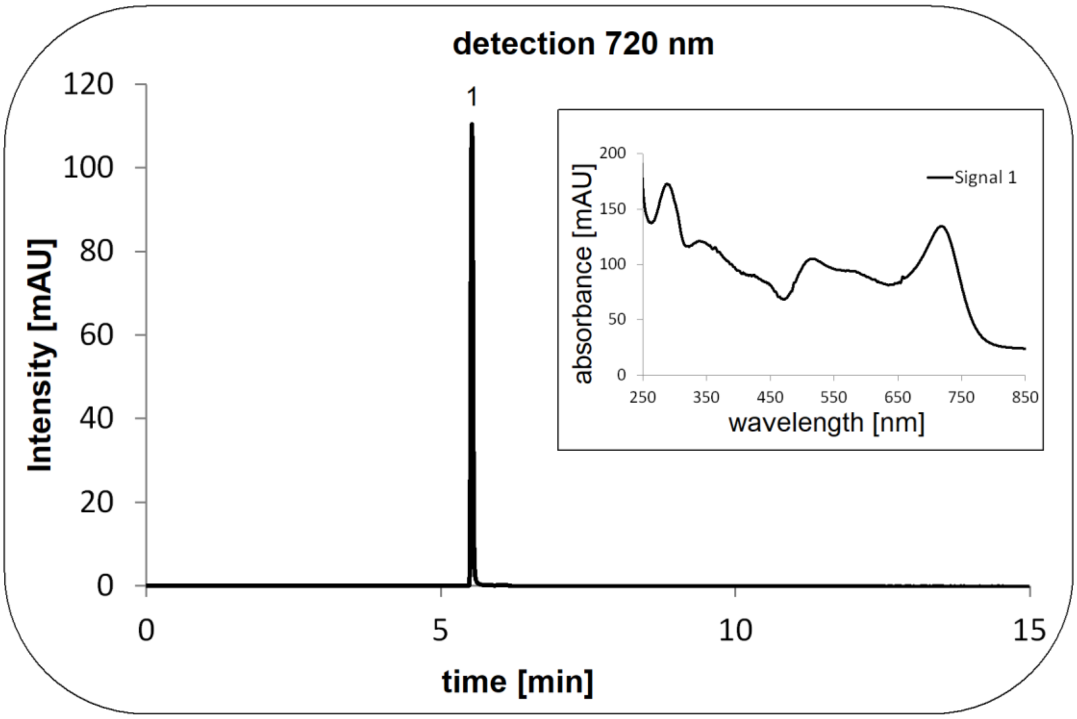

Results			
signal	retention time [min]	area	purity [%]
detection 340 nm			
1	5.5	106.6	100.0
detection 720 nm			
1	5.5	287.9	100.0

Configuration 3

Phase				
time [min]	dichloromethane	acetonitrile	methanol	2-propanol
0	80	20	0	0
3	80	20	0	0
4	60	10	20	10
15	60	10	20	10

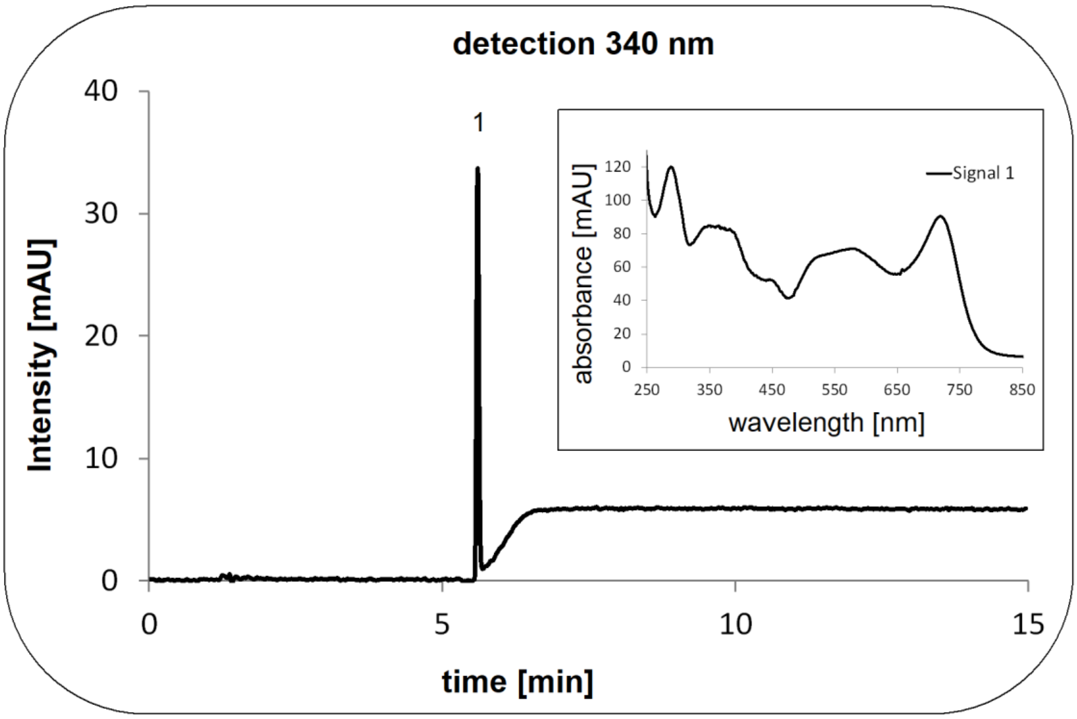

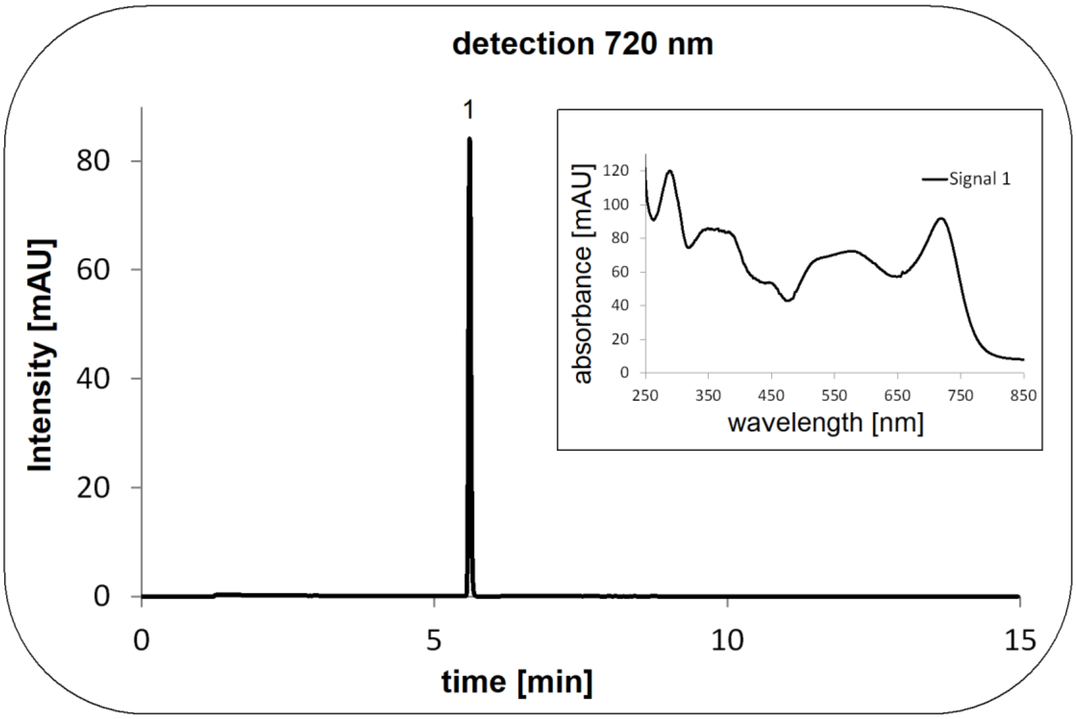

Results			
signal	retention time [min]	area	purity [%]
detection 340 nm			
1	5.6	99.2	100.0
detection 720 nm			
1	5.6	246.6	100.0

2. Electrochemical studies

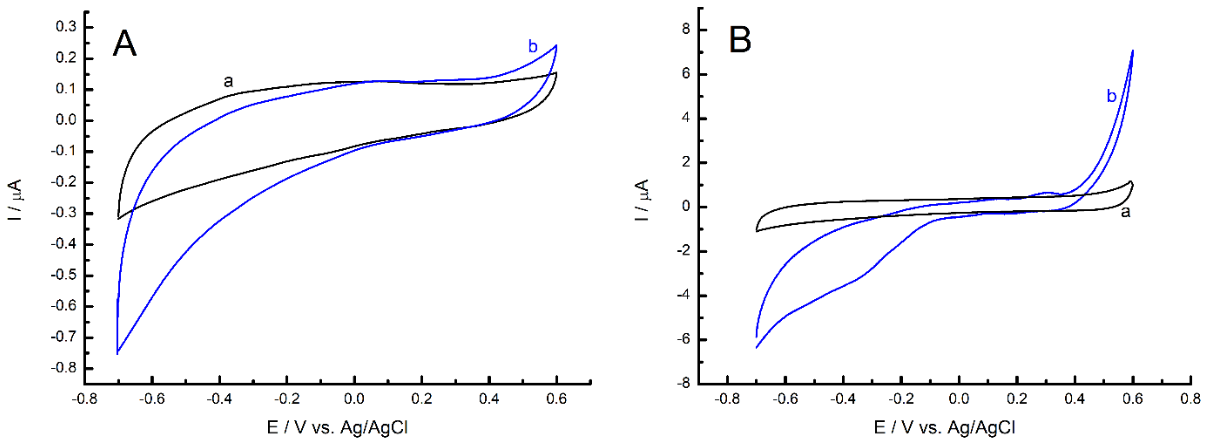

Figure S1. CVs of GC (A), and GC/MWCNT (B) electrodes. An experiment was recorded in PB (pH 7.4) before (a, marked by black line) and after (b, marked by blue line) 2 mM H_2O_2 addition. Scan rate 10 mV s^{-1}.

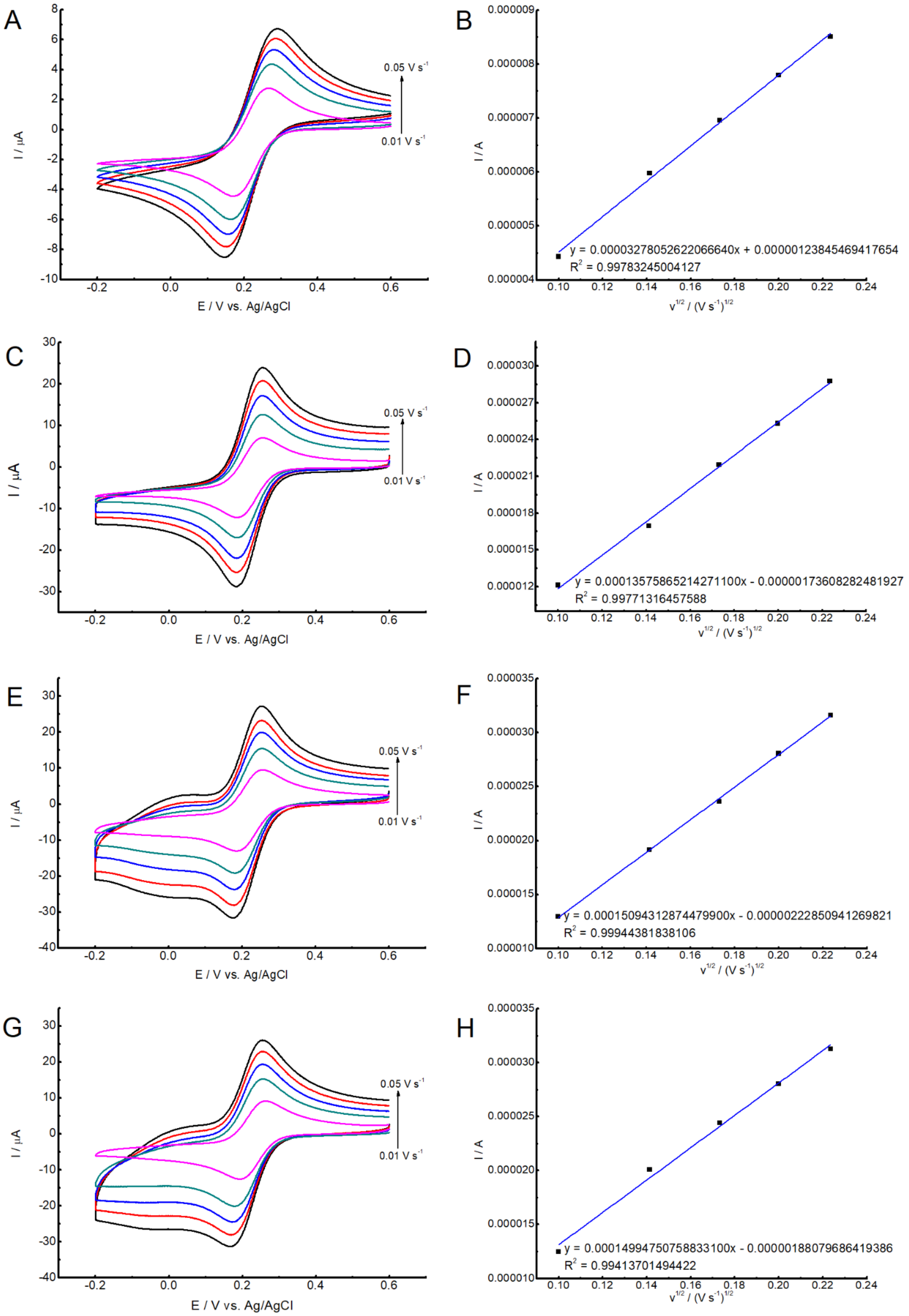

A
I / μA
E / V vs. Ag/AgCl
0.05 V s-1
0.01 V s-1
B
I / A
v1/2 / (V s-1)1/2
y = 0.00003278052622066640x + 0.00000123845469417654
R2 = 0.99783245004127
C
I / μA
E / V vs. Ag/AgCl
0.05 V s-1
0.01 V s-1
D
I / A
v1/2 / (V s-1)1/2
y = 0.00013575865214271100x - 0.00000173608282481927
R2 = 0.99771316457588
E
I / μA
E / V vs. Ag/AgCl
0.05 V s-1
0.01 V s-1
F
I / A
v1/2 / (V s-1)1/2
y = 0.00015094312874479900x - 0.00000222850941269821
R2 = 0.99944381838106
G
I / μA
E / V vs. Ag/AgCl
0.05 V s-1
0.01 V s-1
H
I / A
v1/2 / (V s-1)1/2
y = 0.00014994750758833100x - 0.00000188079686419386
R2 = 0.99413701494422

Figure S2. The results of cyclic voltammetry studies of (A) GC, (C) GC/MWCNT, (E) GC/MWCNT/**Pz3**, and (G) GC/MWCNT/**Pz3**/GOx in phosphate buffer (pH 7.4) containing 1 mM $K_3[Fe(CN)_6]$ at the scan rate range 10–50 mV s^{-1}. (B, D, F, H) Change in peak cathode currents as a function of the applied square root of the scan rate for appropriate electrodes.

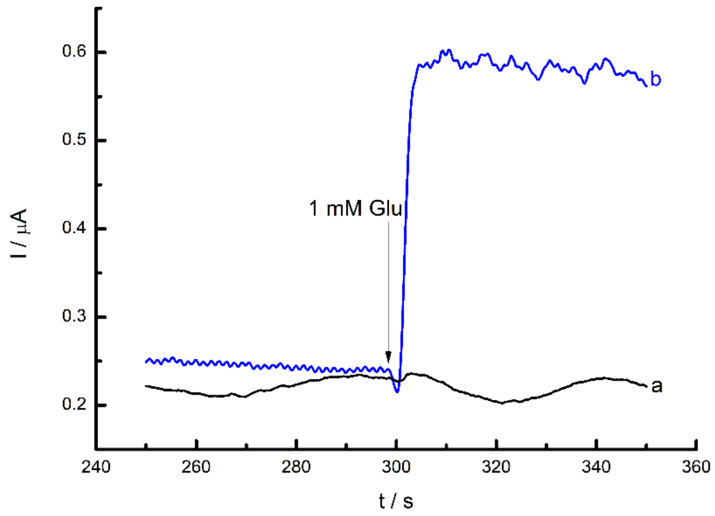

Figure S3. Chronoamperometric response to the addition of 1 mM Glu for the GC/MWCNT/GOx (a, marked by black line), and GC/MWCNT/**Pz3**/GOx (b, marked by blue line) electrode at an applied potential of +0.6 V.

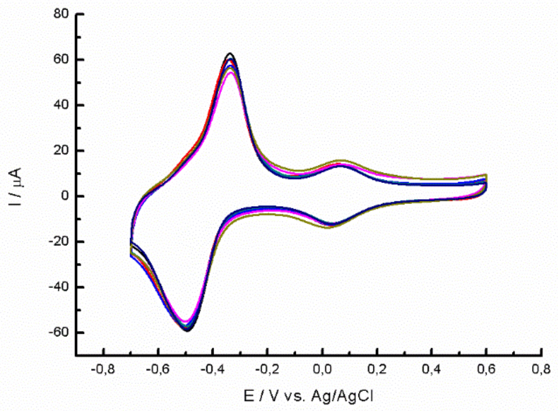

Figure S4. Cyclic voltammograms of the 7 newly prepared GC/MWCNT/**Pz3**/GOx electrodes recorded in PB buffer at 50 mV s^{-1}. RSD is equal 4.6% (n = 7).

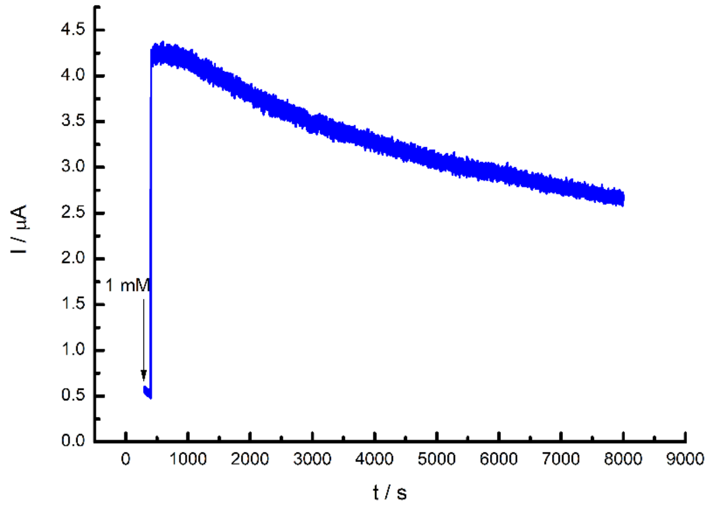

Figure S5. Stability of the GC/MWCNT/**Pz3**/GOx biosensor to a 1 mM Glu response recorded over time with an applied potential of +0.6 V.

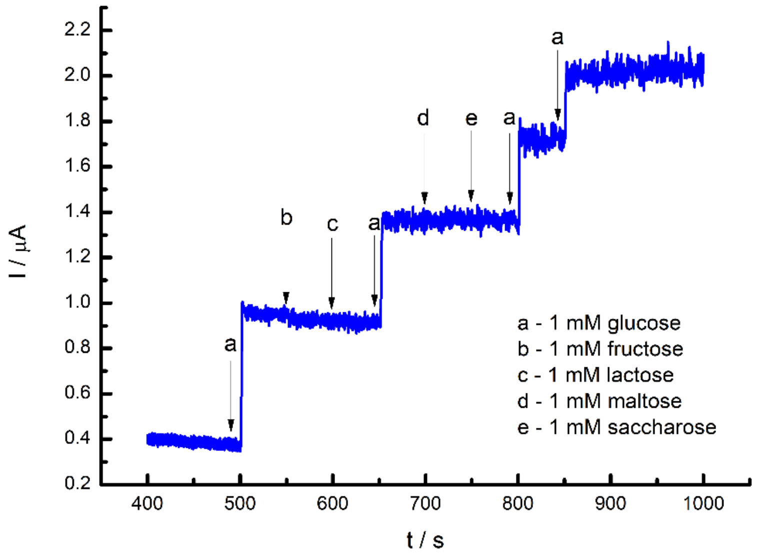

Figure S6. Chronoamperometric responses recorded at the GC/MWCNT/**Pz3**/GOx biosensor upon the addition of glucose and selected interferents with an applied potential of +0.6 V. The supporting electrolyte: phosphate buffer (PB, pH 7.4).

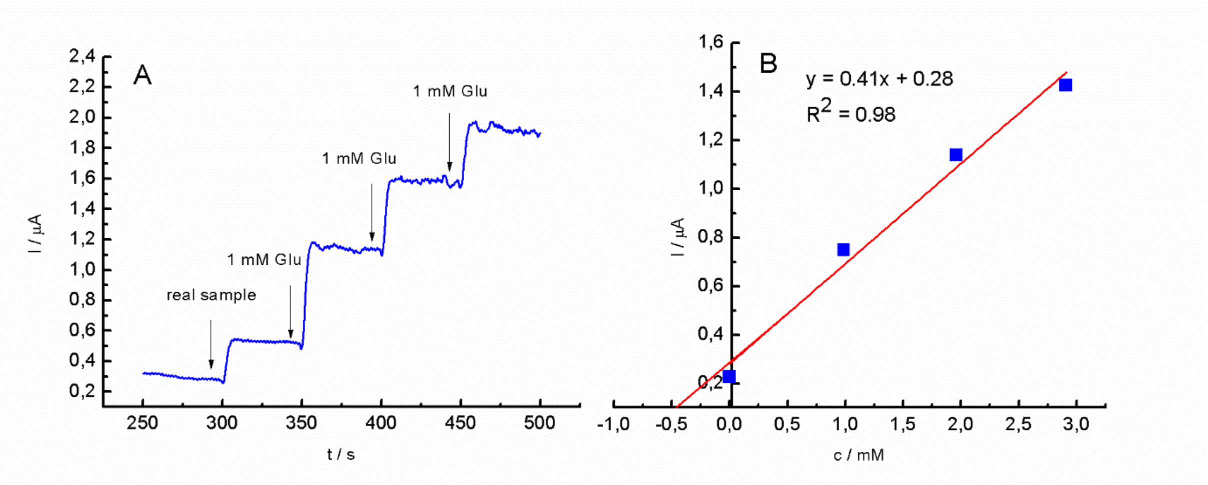

Figure S7. (A) Chronoamperometric curve recorded at 0.6 V in PB after additions of real sample and 1 mM glucose standard solutions. (B) Standard addition curve corresponding to curve presented in (A). RSD = 0.7% (n = 3).
